# Spatial Patterns of a Predator-Prey System of Leslie Type with Time Delay

**DOI:** 10.1371/journal.pone.0150503

**Published:** 2016-03-01

**Authors:** Caiyun Wang, Lili Chang, Huifeng Liu

**Affiliations:** 1 Department of Mathematics, Xinzhou Teachers University, Xinzhou 034000, Shan’xi, China; 2 Complex Systems Research Center, Shanxi University, Taiyuan, Shanxi 030006, China; 3 College of Material Science and Engineering, Taiyuan University of Science and Technology, Taiyuan 030024, Shan’xi, China; Shanxi University, CHINA

## Abstract

Time delay due to maturation time, capturing time or other reasons widely exists in biological systems. In this paper, a predator-prey system of Leslie type with diffusion and time delay is studied based on mathematical analysis and numerical simulations. Conditions for both delay induced and diffusion induced Turing instability are obtained by using bifurcation theory. Furthermore, a series of numerical simulations are performed to illustrate the spatial patterns, which reveal the information of density changes of both prey and predator populations. The obtained results show that the interaction between diffusion and time delay may give rise to rich dynamics in ecosystems.

## Introduction

Thanks to the classical work of Lotka (in 1925) and Volterra (in 1926), modeling predator-prey interaction system has become one of the hot issues in mathematical ecology [1–6]. As is well known, one of the principles that predator-prey models follow is that predators can grow as a function of what they have eaten [7]. One of the famous functional response function is Generalized Holling type III [8]. When *b* = 0, it is called Holling type III. Moreover, not only is the predator growth term described by a function of the prey density, but also is described as a function of the ratio of predator and their prey, *y*/*x*, where *x* and *y* stand for prey and predator density respectively, see for example [9, 10]. The predator-prey system takes the following form:

$$\begin{array}{c} \left\{ \begin{array}{c} \frac{dx}{d\tau }=xg\left(x,K\right)-yp\left(x\right),\\ \frac{dy}{d\tau }=yq\left(\frac{y}{x}\right), \end{array}\right. \end{array}$$ (1)

where *g*(*x*, *K*) describes the specific rate of the prey if there is no predator. *p*(*x*) is the functional response function which describes the change in the density of the prey when they are attacked by per predator in per unit time. Information about the properties of function *g*(*x*, *K*), *p*(*x*) and *q*(*x*) are available in [11, 12]. In this paper, we consider system (1) with the following functions, $g\left(x,K\right)=r\left(x-\frac{x}{K}\right),p\left(x\right)=\frac{mx^{2}y}{ax^{2}+1}$ and $q\left(x\right)=y\theta \left(1-\frac{hy}{x}\right)$, namely, the predator-prey system of Lesile type with Holling type III functional response. Then Eq (1) becomes:

$$\begin{array}{c} \left\{ \begin{array}{c} \frac{dx}{d\tau }=rx(1-\frac{x}{K})-\frac{mx^{2}y}{ax^{2}+1},\\ \frac{dy}{d\tau }=y\theta (1-\frac{hy}{x}), \end{array}\right. \end{array}$$ (2)

where the parameter *r*, *K*, *a*, *θ*, *m* and *h* are all positive constants. *r* is the prey intrinsic growth rate. *K* is the carrying capacity. *m* is capturing rate. *a* is half capturing rate. *θ* is predator intrinsic growth rate. *h* is conversion rate of prey into predator biomass. For the sake of convenience, system (2) should be rewritten into the nondimensional form. Assuming

$$\begin{array}{c} u=\frac{x}{K},\;\;v=\frac{mky}{r},\;\;t=r\tau ,\;\;\eta =\frac{\theta }{r},\;\;\epsilon =ak^{2},\;\;\gamma =\frac{hr}{mk^{2}}, \end{array}$$

then system (2) becomes:

$$\begin{array}{c} \left\{ \begin{array}{c} \frac{du}{dt}=u\left(1-u\right)-\frac{u^{2}v}{\epsilon u^{2}+1}:=f\left(u,v\right),\\ \frac{dv}{dt}=v\eta (1-\frac{\gamma v}{u}):=g\left(u,v\right):=v\eta \left[1-q\left(u,v\right)\right]. \end{array}\right. \end{array}$$ (3)

Pattern formation in reaction-diffusion system is one of the attractive problems in natural, social, and technological sciences. Espatially in ecological system, various predator-prey models with diffusion have been studied [13–18]. Pattern formation can well explain species survival under the influence of individual mobility. Combing Eq (3) with diffusion, we have the spatiotemporal predator-prey system of Lesile type with Holling type III functional response:

$$\begin{array}{c} \left\{ \begin{array}{c} \frac{\partial u}{\partial t}=u\left(1-u\right)-\frac{u^{2}v}{\epsilon u^{2}+1}+D_{1}\nabla^{2}u,\\ \frac{\partial v}{\partial t}=v\eta (1-\frac{\gamma v}{u})+D_{2}\nabla^{2}v, \end{array}\right. \end{array}$$ (4)

where the positive constants *D*_1_ and *D*_2_ denote the diffusive coefficients of *u* and *v*, respectively. $\nabla^{2}=\frac{\partial^{2}}{\partial x^{2}}+\frac{\partial^{2}}{\partial y^{2}}$ is the usual Laplacian operator in two-dimensional space, which describes the random motion.

On the other hand, time delay due to maturation time, capturing time, gestation or other reasons widely exists and plays an important role in many biological dynamical systems [19, 20]. In order to reflect the current population dynamics, the rate of change of which depends on the past population of the system, we should incorporate time delays into mathematical models [21–24]. Although a lot of work has been done about the spatial predator-prey model [25–28] and studies of delay feedback on pattern formation have achieved great progress [29–33], study of delay driven pattern formation in a Leslie type system with Holling type III functional response seems to be rare. As a result, in the present paper we aim to study the effects of time delay on the spatiotemporal dynamics of a Leslie type model with Holling type III functional response. The model is as following form:

$$\begin{array}{c} \left\{ \begin{array}{c} \frac{\partial u}{\partial t}=u\left(1-u\right)-\frac{u^{2}v}{\epsilon u^{2}+1}+D_{1}\nabla^{2}u,\\ \frac{\partial v}{\partial t}=v\eta [1-\frac{\gamma v(t-\tau )}{u(t-\tau )}]+D_{2}\nabla^{2}v, \end{array}\right. \end{array}$$ (5)

where *τ* > 0 is a constant due to the negative feedback. Let Ω be a square flat domain. The initial conditions are

$$\begin{array}{c} u(x,y;t)>0,\;\;v(x,y;t)>0,\;\;\;\;(x,y)\in \Omega =(0,L)\times (0,L)\;\;\;\;with\;\;\;\;t\in [-\tau ,0]. \end{array}$$ (6)

Generally speaking, to make sure that Turing pattern is determined by reaction-diffusion mechanism, we usually choose zero-flux boundary conditions

$$\begin{array}{c} \frac{\partial u}{\partial n}{|}_{\left(x,y\right)}=\frac{\partial v}{\partial n}{|}_{\left(x,y\right)}=0, \end{array}$$ (7)

which means that there is no flux of populations through the boundary, i.e., no external input is imposed from outside.

This paper is organized as follows. In section 2, we study the dynamics of model without delay. In section 3, we obtain the condition of Turing instability (diffusion and delay induced instabilities)via linear stability analysis. In section 4, we present various spatial patterns by performing numerical simulations. Finally, we give some conclusions and discussions in section 5.

## Materials and Methods

### Existence of positive equilibria

We need to analyze the stability criteria of model (5) without delay and diffusion. The corresponding model is system (3). Obviously, system (3) has equilibrium *E*_0_ = (1, 0), which corresponds to extinction of the predator. From the biological point of view, we are interested in the interior equilibria points, which are the positive solutions of the following cubic polynomial equations of the system (3):

$$\begin{array}{c} \left\{ \begin{array}{c} \left(1-u\right)-\frac{uv}{\epsilon u^{2}+1}=0,\\ v=\frac{u}{\gamma }. \end{array}\right. \end{array}$$ (8)

Substituting the second equality for the first equality in Eq (8), we have that:

$$\begin{array}{c} F\left(u\right):=u^{3}+\omega_{2}u^{2}+\omega_{1}u+\omega_{0}=0, \end{array}$$ (9)

where $\omega_{2}=\frac{1}{\gamma \epsilon }-1,\omega_{1}=\frac{1}{\epsilon }$ and $\omega_{0}=-\frac{1}{\epsilon }<0$. The number of equilibria in Eq (3) is determined by the number of real roots of *F* in the interval *I*_0_ = (0, 1). In addition, *F*′(*u*) = 3*u*^2^ + 2*ω*_2_*u* + *ω*_1_ has two zeros

$$\begin{array}{c} \xi_{\pm }=\frac{\gamma \epsilon -1\pm \sqrt{\Delta_{1}}}{3\epsilon \gamma }, \end{array}$$ (10)

when

$$\begin{array}{c} \Delta_{1}:={\left(\gamma \epsilon -1\right)}^{2}-3\epsilon \gamma^{2}\geq 0. \end{array}$$ (11)

The discriminant of the cubic polynomial *F* is given by

$$\begin{array}{c} \Delta_{2}:={\left(\frac{Q}{2}\right)}^{2}+{\left(\frac{P}{3}\right)}^{3}, \end{array}$$ (12)

where $P=\omega_{1}-\omega_{2}^{2}/3$ and $Q=(2\omega_{2}^{3}-9\omega_{1}\omega_{2}+27\omega_{0})/27$. Similar to Lemma 1 in [34], we have the following results.

System (3) has at least one equilibrium and at most three equilibria in the interval *I*_0_ = (0, 1). Moreover, consider the following condition:

$$\begin{array}{c} 0<\xi_{\pm }<1; \end{array}$$ (13)

$$\begin{array}{c} F\left(\xi_{-}\right)>0\;\;and\;\;F\left(\xi_{+}\right)<0; \end{array}$$ (14)

$$\begin{array}{c} \Delta_{2}=0; \end{array}$$ (15)

$$\begin{array}{c} 0<2\sqrt[{3}]{\pm Q/2}-\gamma^{2}/3<1; \end{array}$$ (16)

$$\begin{array}{c} F\left(\xi_{-}\right)F\left(\xi_{+}\right)>0; \end{array}$$ (17)

$$\begin{array}{c} \xi_{\pm }<0. \end{array}$$ (18)

1. If and only if Eqs (13) and (14) hold, then system (3) has three equilibria in the interval *I*_0_ = (0, 1).
2. If and only if Eqs (15) and (16) hold, system (3) two equilibria in the interval *I*_0_ = (0, 1).
3. If and only if Eqs (18), or(13) and (17) hold, system (3) has a unique equilibrium in the interval *I*_0_ = (0, 1) Moreover, if system (3) has a unique equilibrium $E_{1}\left(u_{1}^{*},v_{1}^{*}\right)$ where $v_{1}^{*}=u_{1}^{*}/\gamma$, then $u_{1}^{*}$ is either a simple zero of *F* or a zero of multiplicity 3 of *F*.

We describe qualitative properties and stability of the interior equilibria of system (3) (see S1 File). By the proof of **Theorem 1** (see S1 File), we have the following conclusion.

**Fig 1:**
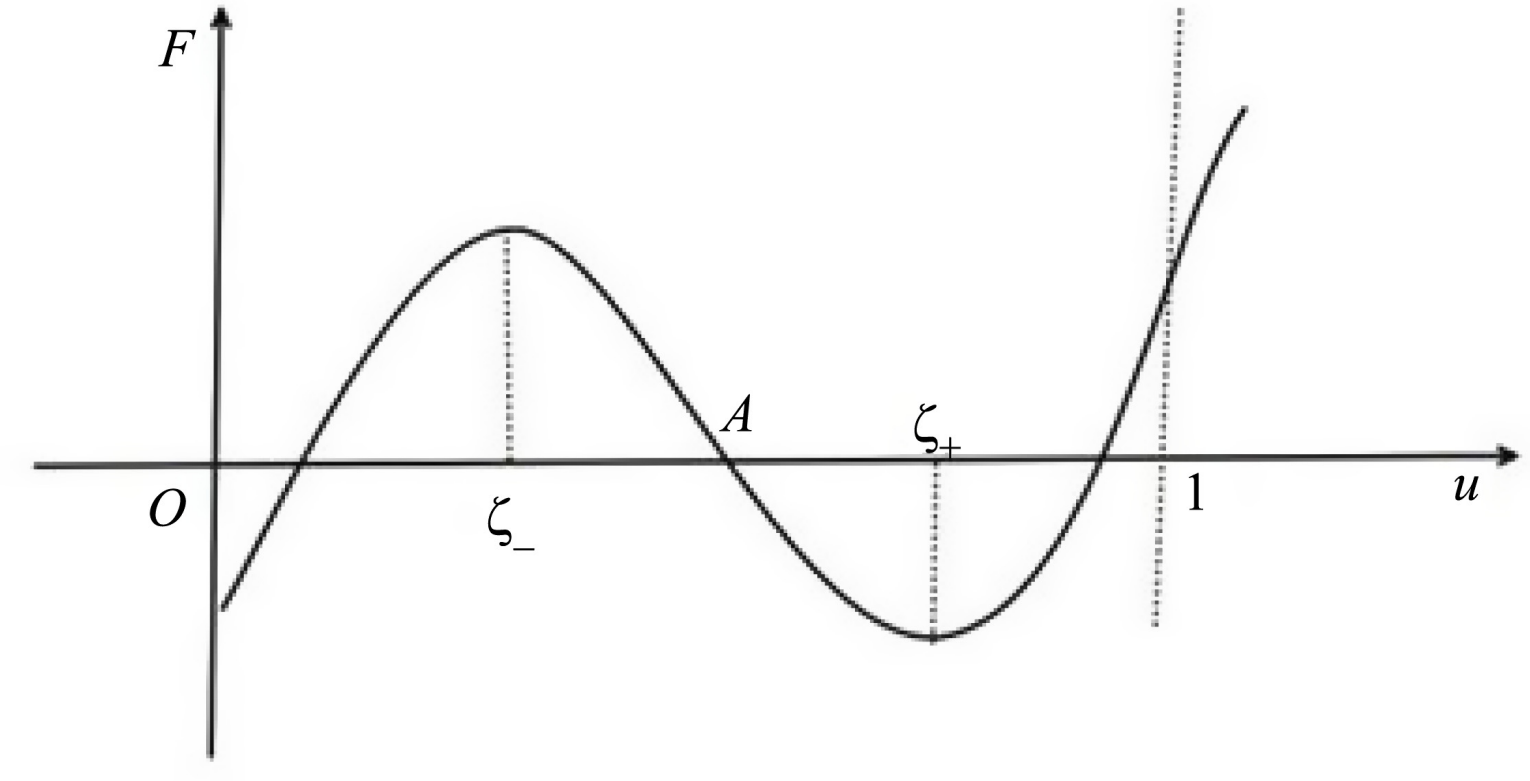
*F* has three zero solutions in *I*_0_.

**Fig 2:**
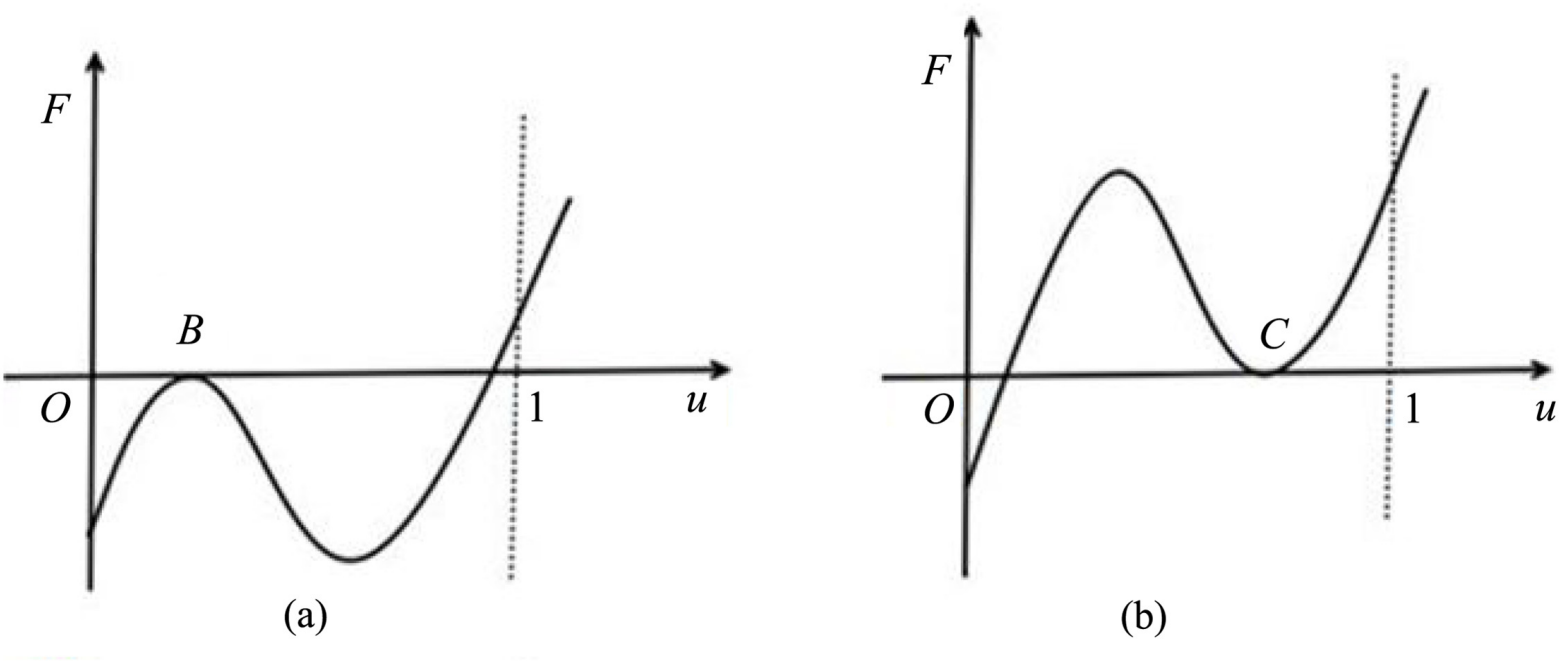
*F* has two zero solutions in *I*_0_.

**Fig 3:**
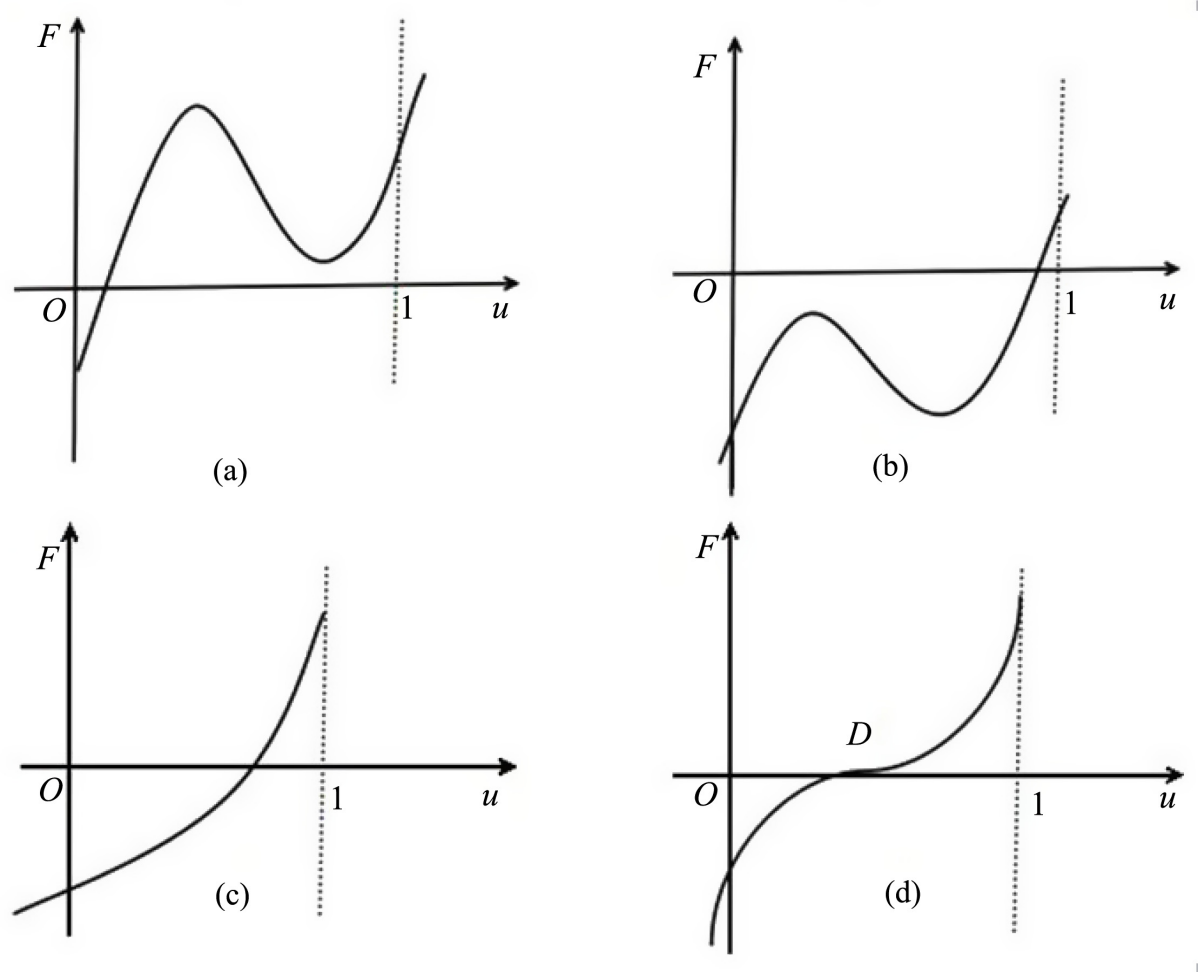
*F* has a unique zero solution in *I*_0_.

If

$$\begin{array}{c} \text{tr}\left(J\right)=f_{u}+g_{v}<0, \end{array}$$ (19)

all of the positive equilibria of system (3) are stable, except the saddle (corresponds to the zero *A* of *F* in Fig 1) and degenerate equilibria (corresponds to the zeros *B*, *C* and *D* of *F* in Figs 2 and 3 respectively).

### Turing instability analysis of model (4)

In this subsection, we will give a brief analysis of Turing instability of model (4). For the sake of convenience, let E*(u*, v*) be anyone of the interior steady equilibrium in **Theorem 1** (see S1 File). The characteristic polynomial at E*(u*, v*) is

$$\begin{array}{c} |\lambda E-J_{k}|=0, \end{array}$$ (20)

where *J*_*k*_ = *J* − *diag*(*D*_1_, *D*_2_)*k*^2^, *K* is a wavenumber and *J* is the Jacobian matrix of system (3) at E*(u*, v*).

Further, Eq (20) yields

$$\begin{array}{c} \lambda^{2}-\text{tr}\left(J_{k}\right)\lambda +\text{det}\left(J_{k}\right)=0, \end{array}$$ (21)

where

$$\begin{array}{c} \text{tr}\left(J_{k}\right)=\text{tr}\left(J\right)-\left(D_{1}+D_{2}\right)k^{2}, \end{array}$$ (22)

and

$$\begin{array}{c} \text{det}\left(J_{k}\right)=\text{det}\left(J\right)+D_{1}D_{2}k^{4}-\left(D_{2}f_{u}+D_{1}g_{v}\right)k^{2}. \end{array}$$ (23)

We can get the roots of Eq (20):

$$\begin{array}{c} \lambda_{k}=\frac{\text{tr}\left(J_{k}\right)\pm \sqrt{\text{tr}{\left(J_{k}\right)}^{2}-4\text{det}\left(J_{k}\right)}}{2}. \end{array}$$ (24)

The condition for the onset of Hopf instability holds when a pair of imaginary eigenvalues cross the real axis from the negative value to the positive one and there is no diffusion [35, 36]. That is to say, the Hopf bifurcation occurs when

$$\begin{array}{c} Im\left(\lambda_{k}\right)\neq 0,\;\;\;\;Re\left(\lambda_{k}\right)=0\;\;\;\;\;at\;\;k=0. \end{array}$$

A homogeneously steady state is said to be Turing instability if it is stable for model (3) without diffusion but becomes unstable because of homogeneous perturbation caused by diffusion. A general linear analysis [37–39] show that the necessary conditions for onset of Turing instability for model (4) are given by condition Eq (19) and the following conditions

$$\begin{array}{c} \text{det}\left(J\right)=f_{u}g_{v}-f_{v}g_{u}>0, \end{array}$$ (25)

$$\begin{array}{c} D_{2}f_{u}+D_{1}g_{v}>0, \end{array}$$ (26)

$$\begin{array}{c} {\left(D_{2}f_{u}+D_{1}g_{v}\right)}^{2}>4D_{1}D_{2}\left(f_{u}g_{v}-f_{v}g_{u}\right). \end{array}$$ (27)

The condition Eqs (19) and (25) make sure that the equilibrium E* = (u*, v*) is stable for model (3) without diffusion, and becomes unstable for model (4) if *Re*(*λ*_*k*_) transits the real axis from a negative side to a positive one (corresponding to condition Eq (26) and (27)). Namely, the Turing bifurcation occurs when

$$\begin{array}{c} Im\left(\lambda_{k}\right)=0,\;\;\;\;Re\left(\lambda_{k}\right)=0\;\;\;\;\;at\;\;k=k_{T}\neq 0, \end{array}$$

and wavenumber *k*_*T*_ satisfies

$$\begin{array}{c} k_{T}^{2}=\sqrt{\frac{\text{det}(J)}{D_{1}D_{2}}}. \end{array}$$

## Results

### Linear stability analysis

In this subsection, we will consider the stability of model (5). Obviously, model (5) has the same equilibria as model (4). Similar to [30, 31], assume that *τ* is small enough, then we replace $u\left(x,y,t-\tau \right)=u\left(x,y,t\right)-\tau \frac{\partial u(x,y,t)}{\partial t}$ and $v\left(x,y,t-\tau \right)=v\left(x,y,t\right)-\tau \frac{\partial v(x,y,t)}{\partial t}$ in system (5) and obtain the following equations:

$$\begin{array}{c} \left\{ \begin{array}{c} \frac{\partial u}{\partial t}=f\left(u,v\right)+D_{1}\nabla^{2}u,\\ \frac{\partial v}{\partial t}=v\eta \left[1-q(u\left(x,y,t\right)-\tau \frac{\partial u(x,y,t)}{\partial t},v\left(x,y,t\right)-\tau \frac{\partial v(x,y,t)}{\partial t})\right]+D_{2}\nabla^{2}v. \end{array}\right. \end{array}$$ (28)

Expanding Eq (28) in Taylor Series and neglecting the higher order non-linearities, then Eq (28) becomes:

$$\begin{array}{c} \left\{ \begin{array}{c} \frac{\partial u}{\partial t}=f\left(u,v\right)+D_{1}\nabla^{2}u,\\ \frac{\partial v}{\partial t}=v\eta \left[1-q\left(u,v\right)+\tau q_{u}\frac{\partial u(x,y,t)}{\partial t}+\tau q_{v}\frac{\partial v(x,y,t)}{\partial t}\right]+D_{2}\nabla^{2}v, \end{array}\right. \end{array}$$ (29)

where $q_{u}=\frac{\partial q}{\partial u}$ and $q_{v}=\frac{\partial q}{\partial v}$. By Eq (29), we obtain the following equations:

$$\begin{array}{c} \left\{ \begin{array}{c} \frac{\partial u}{\partial t}=f\left(u,v\right)+D_{1}\nabla^{2}u,\\ \frac{\partial v}{\partial t}=\frac{1}{1-v\eta \tau q_{v}}g\left(u,v\right)+\frac{v\eta \tau q_{u}}{1-v\eta \tau q_{v}}f\left(u,v\right)+\frac{v\eta \tau q_{u}}{1-v\eta \tau q_{v}}D_{1}\nabla^{2}u+\frac{1}{1-v\eta \tau q_{v}}D_{2}\nabla^{2}v. \end{array}\right. \end{array}$$ (30)

If we take small spatiotemporal perturbations *δu*(*x*, *y*, *t*) and *δv*(*x*, *y*, *t*) on the steady state E* = (u*, v*) of system (5), then we have:

$$\begin{array}{c} u\left(x,y,t\right)=u^{*}+\delta u\left(x,y,t\right),\;\;\;\;\;v\left(x,y,t\right)=v^{*}+\delta v\left(x,y,t\right). \end{array}$$ (31)

Expanding the reaction terms around the steady state E* = (u*, v*) in Taylor Series up to first order and rearranging the terms, we obtain:

$$\begin{array}{c} \left\{ \begin{array}{c} \frac{\partial (\delta u)}{\partial t}=f_{u}\left(\delta u\right)+f_{v}\left(\delta v\right)+D_{1}\nabla^{2}\left(\delta u\right),\\ \frac{\partial (\delta v)}{\partial t}=(\frac{g_{u}}{1-v^{*}\eta \tau q_{v}}+\frac{v^{*}\eta \tau q_{u}f_{u}}{1-v^{*}\eta \tau q_{v}})\left(\delta u\right)+\left(\frac{g_{v}}{1-v^{*}\eta \tau q_{v}}+\frac{v^{*}\eta \tau q_{u}f_{v}}{1-v^{*}\eta \tau q_{v}}\right)\left(\delta v\right)\\ \;\;\;\;\;\;\;\;\;\;\;\;+\frac{v^{*}\eta \tau q_{u}D_{1}\nabla^{2}\left(\delta u\right)}{1-v^{*}\eta \tau q_{v}}+\frac{D_{2}\nabla^{2}\left(\delta v\right)}{1-v^{*}\eta \tau q_{v}}, \end{array}\right. \end{array}$$ (32)

where $f_{u}={\frac{\partial f}{\partial u}|}_{\left(u^{*}v^{*}\right)}f_{v}={\frac{\partial f}{\partial v}|}_{\left(u^{*},v^{*}\right)},g_{u}={\frac{\partial g}{\partial u}|}_{\left(u^{*},v^{*}\right)},g_{v}={\frac{\partial q}{\partial v}|}_{\left(u^{*},v^{*}\right)},q_{u}={\frac{\partial q}{\partial u}|}_{\left(u^{*},v^{*}\right)},q_{v}={\frac{\partial q}{\partial v}|}_{\left(u^{*},v^{*}\right)},$ with ${\frac{1}{1-v^{*}\eta \tau q_{v}}|}_{\left(u^{*},v^{*}\right)}=\frac{1}{1-\eta \tau }≜\chi$. Since *τ* is small, we only consider $\tau <\frac{1}{d}\left(i.e.\;\chi >0\right)$ in this paper. Model (32) becomes:

$$\begin{array}{c} \left\{ \begin{array}{c} \frac{\partial (\delta u)}{\partial t}=f_{u}\left(\delta u\right)+f_{v}\left(\delta v\right)+D_{1}\nabla^{2}\left(\delta u\right),\\ \frac{\partial (\delta v)}{\partial t}=\chi (g_{u}-\frac{\eta \tau f_{u}}{\gamma })\left(\delta u\right)+\chi (g_{v}-\frac{\eta \tau f_{u}}{\gamma })\left(\delta v\right)-\frac{\chi \eta \tau }{\gamma }D_{1}\nabla^{2}\left(\delta u\right)+\chi D_{2}\nabla^{2}\left(\delta v\right). \end{array}\right. \end{array}$$ (33)

Assume that spatiotemporal perturbations *δu*(*x*, *y*, *t*) and *δv*(*x*, *y*, *t*) take the following form:

$$\begin{array}{c} \delta u\left(x,y,t\right)=\delta u^{*}e^{\lambda t}\cos k_{x}x\cos k_{y}y,\;\;\;\;\;\;\delta v\left(x,y,t\right)=\delta v^{*}e^{\lambda t}\cos k_{x}x\cos k_{y}y, \end{array}$$ (34)

where *λ* is the growth rate of the perturbation in time *t*, δu* and δv* stand for the amplitudes, and *k*_*x*_ and *k*_*y*_ are the wavenumbers of the solutions. Inserting Eqs (34) into (33), we obtain the characteristic equation at E* = (u*, v*) of model (5):

$$\begin{array}{c} \text{det}\left(\lambda E-J_{\tau k}\right)=\lambda^{2}-\text{tr}\left(J_{\tau k}\right)\lambda +\text{det}\left(J_{\tau k}\right)=0, \end{array}$$ (35)

where

$$\begin{array}{c} J_{\tau k}=(\begin{array}{cc} f_{u}-D_{1}k^{2} & f_{v}\\ \chi \left(g_{u}-\frac{\eta \tau f_{u}}{\gamma }\right)+\frac{\eta \tau \chi D_{1}k^{2}}{\gamma } & \chi \left(g_{v}-\frac{\eta \tau f_{v}}{\gamma }\right)-\chi D_{2}k^{2} \end{array}), \end{array}$$ (36)

$$\begin{array}{c} \text{tr}\left(J_{\tau k}\right)=f_{u}+\chi \left(g_{v}-\frac{\eta \tau f_{u}}{\gamma }\right)-\left(\chi D_{2}+D_{1}\right)k^{2}, \end{array}$$ (37)

$$\begin{array}{c} \text{det}\left(J_{\tau k}\right)=\chi D_{1}D_{2}k^{4}-\chi \left(D_{1}g_{v}+D_{2}f_{u}\right)k^{2}+\chi \left(f_{u}g_{v}-f_{v}g_{u}\right). \end{array}$$ (38)

Now we are interested in investigating the effects of time delay and diffusion on the dynamical system (5), and we want to know under what conditions for time delay to destabilize the steady state and let the spatiotemporal instability occur. The onset of Turing instability requires at least one of tr(*J*_*τk*_) < 0 and det(*J*_*τk*_) > 0 is violated. So we consider the emergence of the Turing instability in the following two cases:

1. det(*J*_*τk*_) > 0 is violated.
2. tr(*J*_*τk*_) < 0 is violated.

### Diffusion induced instability

In this part, we will consider the first case, i.e. det(*J*_*k*_) > 0 is violated, namely, det(*J*_*τk*_) < 0. It can be seen from Eq (38) that det(*J*_*τk*_) = χ det(*J*_*k*_) and $\chi >0(\tau <\frac{1}{\eta })$, so the sign of det(*J*_*τk*_) is the same as det(*J*_*k*_). From Eq (23), det(*J*_*k*_) < 0 is equivalent to:

$$\begin{array}{c} D_{2}f_{u}+D_{1}g_{v}>0, \end{array}$$ (39)

$$\begin{array}{c} {\left(D_{2}f_{u}+D_{1}g_{v}\right)}^{2}>4D_{1}D_{2}\left(f_{u}g_{v}-f_{v}g_{u}\right), \end{array}$$ (40)

at the critical value of wavenumber $k_{c}^{2}=\frac{D_{1}f_{u}+D_{2}g_{v}}{2D_{1}D_{2}}>0$.

In addition, tr(*J*_*τk*_) < 0 equals to $f_{u}+\chi \left(g_{v}-\frac{\tau \eta f_{v}}{\gamma }\right)<0$. Because that $-\frac{\eta f_{v}}{\gamma }-f_{u}\eta =\text{det}\left(J\right)>0$, simple algebraic computation leads to:

$$\begin{array}{c} 0\leq \tau <-\frac{f_{u}+g_{v}}{-\frac{\eta f_{v}}{\gamma }-f_{u}\eta }≜\tau_{c}. \end{array}$$ (41)

Hence, in this case, *τ* must satisfy

$$\begin{array}{c} \tau <\min(\tau_{c},\frac{1}{\eta }). \end{array}$$ (42)

Finally, we can get the condition of diffusion induced instability:

1. When system (3) has three equilibria, if condition Eqs (11), (13), (14), (19), (39), (40), and (42) hold, then instability of model (5) induced by diffusion occurs.
2. When system (3) has two equilibria, if condition Eqs (11), (15), (16), (19), (39), (40) and (42) hold, then instability of model (5) induced by diffusion occurs.
3. When system (3) has a unique equilibrium, if condition Eqs (11), (18) (or (13) and (17)), (19), (39), (40) and (42) hold, then instability of model (5) induced by diffusion occurs.

### Delay induced instability

In this part, we will consider the seconde case, i.e. tr(*J*_*τk*_) < 0 is violated. In order to find suitable values of parameters for our simulation, we assume $\chi <0(\tau >\frac{1}{\eta })$ in this part. Following the same analysis in subsection B, tr(*J*_*τk*_) < 0 is violated when $f_{u}+\chi \left(g_{v}-\frac{\tau \eta f_{v}}{\gamma }\right)>0$, which yields

$$\begin{array}{c} \tau <-\frac{f_{u}+g_{v}}{-\frac{\eta f_{v}}{\gamma }-f_{u}\eta }≜\tau_{c}. \end{array}$$ (43)

Hence, in this case, *τ* must satisfy:

$$\begin{array}{c} \frac{1}{\eta }<\tau <\tau_{c}. \end{array}$$ (44)

We mainly consider the delay induced instability, therefore we keep det(*J*_*τk*_) > 0, which is equivalent to the same condition as Eqs (39) and (40).

At last, we can get the condition of delay induced instability:

1. When system (3) has three equilibria, if condition Eqs (11), (13), (14), (19), (39), (40) and (44) hold, then instability of model (5) induced by delay occurs.
2. When system (3) has two equilibria, if condition Eqs (11), (15), (16), (19), (39), (40) and (44) hold, then instability of model (5) induced by delay occurs.
3. When system (3) has a unique equilibrium, if condition Eqs (11), (18) (or (13) and (17)), (19), (39), (40) and (44) hold, then instability of model (5) induced by delay occurs.

In this section, we will perform numerical simulations for model (5) on the 100 × 100 square lattices with Neumann boundary conditions. The simulations are initiated with small amplitude random perturbations around the positive equilibrium point *E*(u*, v*). The reaction-diffusion equations in our models are analyzed numerically employing Forward Difference implicit difference scheme. We set the time step Δ*t* = 0.005, spatial mesh size *h* = 1.

We run the simulations until they reach a stationary state which indicates that the behavior does not seem to change its characteristic anymore. As a result, we only make analysis of pattern formation to one distribution(in this paper, we show the distribution of the predator).

### Numerical results

#### A. Pattern formation induced by diffusion

**Fig 4:**
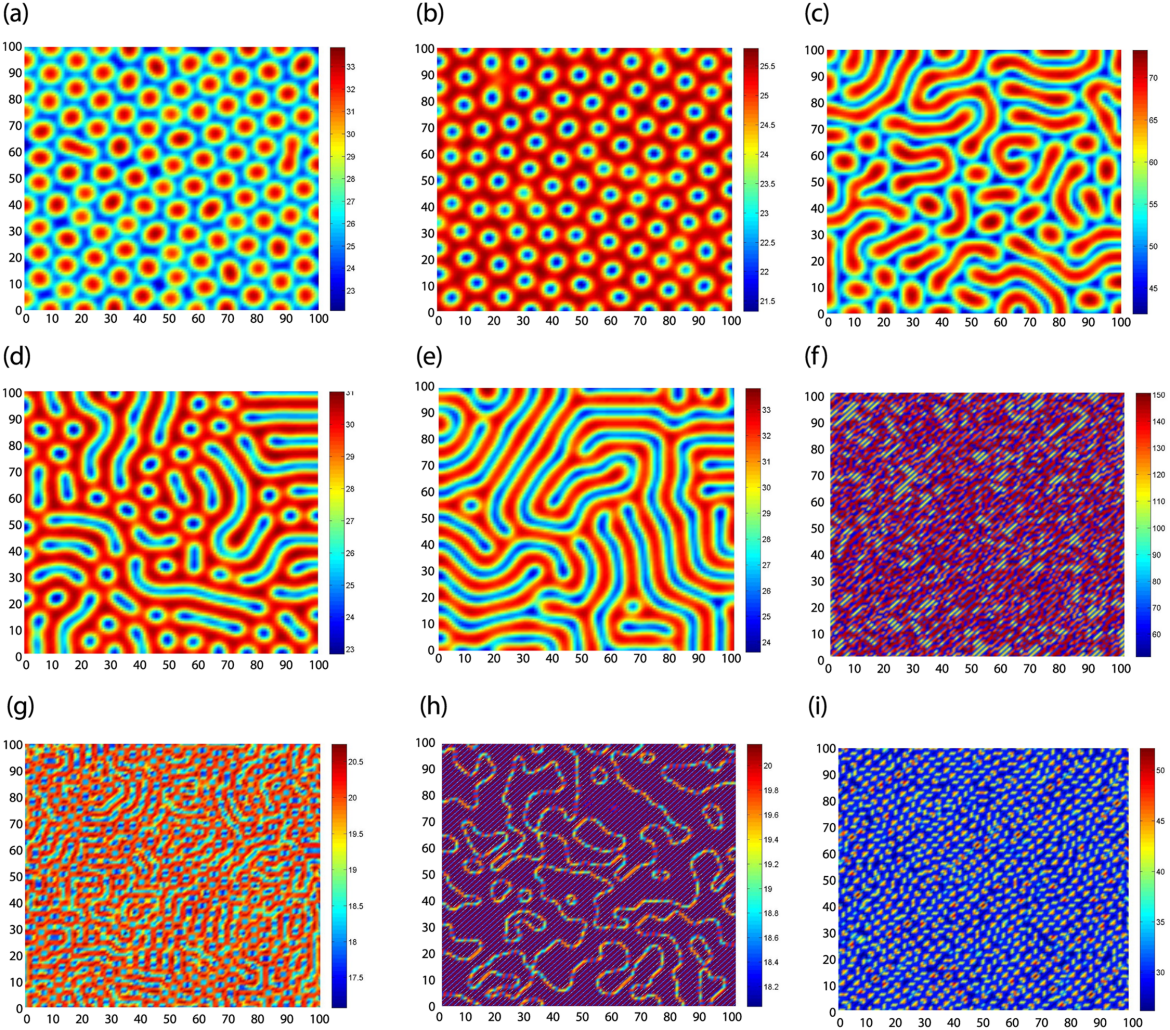
Nine types of patterns induced by diffusion.

In this subsection, we will focus on the pattern formation induced by diffusion, we obtain nine different types of patterns in Fig 4, where prey and predator can coexist.

In Fig 4(a) we take the parameters as

$$\begin{array}{cc} & \eta =0.6,\;\;\tau =0.4,\;\;D_{1}=0.2,\;\;D_{2}=8,\\ & u^{*}=0.14,\;\;v^{*}=22.39685714,\;\;\epsilon =135,\;\;\gamma =0.006250877036. \end{array}$$ (45)

Fig 4(a) is the typical spot pattern, which shows that the distribution of the predator *v* is the isolated regions with high density.

In Fig 4(b) we take the parameters as

$$\begin{array}{cc} & \eta =0.6,\;\;\tau =0.4,\;\;D_{1}=0.2,\;\;D_{2}=8,\\ & u^{*}=0.35,\;\;v^{*}=24.60714285,\;\;\epsilon =100,\;\;\gamma =0.01422351234. \end{array}$$ (46)

Fig 4(b) is the typical hole pattern, which shows that the distribution of the predator *v* is the isolated regions with low density.

In Fig 4(c) we take the parameters as

$$\begin{array}{cc} & \eta =0.6,\;\;\tau =0.4,\;\;D_{1}=0.2,\;\;D_{2}=8,\\ & u^{*}=0.11,\;\;v^{*}=36.48190910,\;\;\epsilon =290,\;\;\gamma =0.003015193084. \end{array}$$ (47)

Fig 4(c) is the mixture of red stripes and red spots named as mixed pattern 1.

In Fig 4(d) we take the parameters as

$$\begin{array}{cc} & \eta =0.6,\;\;\tau =0.4,\;\;D_{1}=0.2,\;\;D_{2}=8,\\ & u^{*}=0.3,\;\;v^{*}=27.53333333,\;\;\epsilon =120,\;\;\gamma =0.01089588378. \end{array}$$ (48)

Fig 4(d) is the mixture of blue stripes and blue spots named as mixed pattern 2.

In Fig 4(e) we take the parameters as

$$\begin{array}{cc} & \eta =0.6,\;\;\tau =0.4,\;\;D_{1}=0.2,\;\;D_{2}=8,\\ & u^{*}=0.25,\;\;v^{*}=27.37500000,\;\;\epsilon =130,\;\;\gamma =0.009132420091. \end{array}$$ (49)

Fig 4(e) is the stripe pattern.

In Fig 4(f) we take the parameters as

$$\begin{array}{cc} & \eta =0.6,\;\;\tau =0.4,\;\;D_{1}=0.01,\;\;D_{2}=0.3,\\ & u^{*}=0.2,\;\;v^{*}=100.00000000,\;\;\epsilon =600,\;\;\gamma =0.00200000. \end{array}$$ (50)

Fig 4(f) is somewhat like labyrinth pattern.

In Fig 4(g) we take the parameters as

$$\begin{array}{cc} & \eta =0.6,\;\;\tau =0.4,\;\;D_{1}=0.04,\;\;D_{2}=0.16,\\ & u^{*}=0.3,\;\;v^{*}=19.13333333,\;\;\epsilon =80,\;\;\gamma =0.01567944251. \end{array}$$ (51)

Fig 4(g) is somewhat like grid pattern.

In Fig 4(h) we take the parameters as

$$\begin{array}{cc} & \eta =0.6,\;\;\tau =0.4,\;\;D_{1}=0.01,\;\;D_{2}=0.33,\\ & u^{*}=0.3,\;\;v^{*}=19.13333333,\;\;\epsilon =80,\;\;\gamma =0.01567944251. \end{array}$$ (52)

Fig 4(h) is the composed parallel lines.

In Fig 4(i) we take the parameters as

$$\begin{array}{cc} & \eta =0.6,\;\;\tau =0.4,\;\;D_{1}=0.02,\;\;D_{2}=0.54,\\ & u^{*}=0.11,\;\;v^{*}=27.67090909,\;\;\epsilon =200,\;\;\gamma =0.003975294040. \end{array}$$ (53)

Fig 4(i) is somewhat butterfly-like patten.

#### B. Pattern formation induced by delay

**Fig 5:**
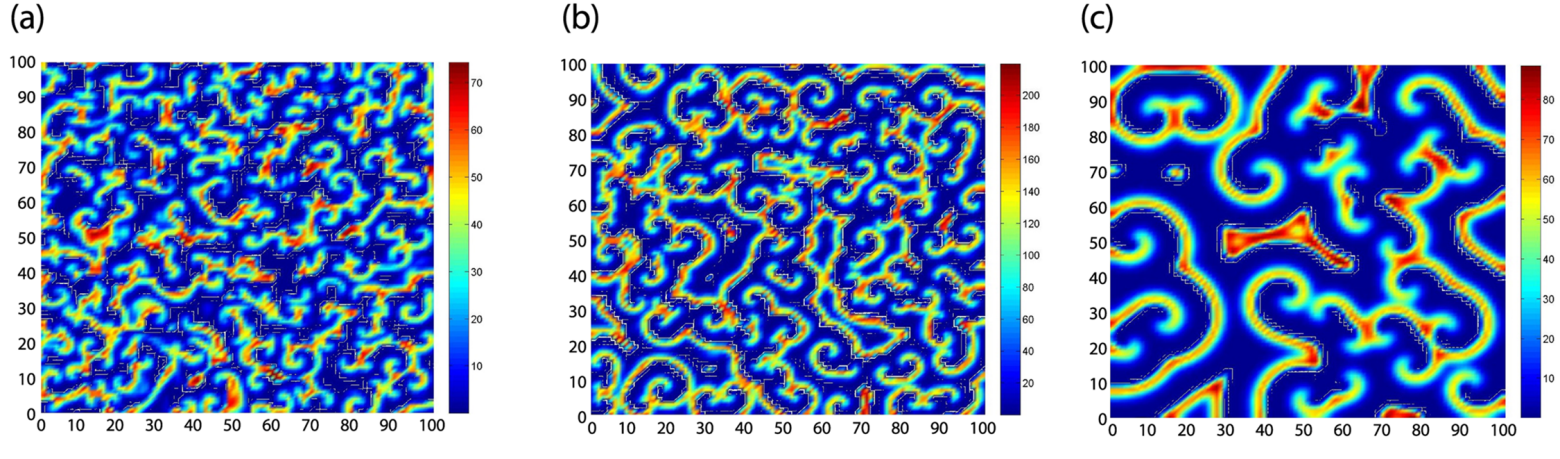
Three types of spirals induced by delay.

In this subsection, we will focus on the pattern formation induced by delay, we obtain three different types of patterns in Fig 5.

In Fig 5(a) we take the parameters as

$$\begin{array}{cc} & \eta =1,\;\;\tau =1.2,\;\;D_{1}=0.002,\;\;D_{2}=0.1,\\ & u^{*}=0.3,\;\;v^{*}=19.13333333,\;\;\epsilon =80,\;\;\gamma =0.01567944251. \end{array}$$ (54)

Fig 5(a) is spirals 1.

In Fig 5(b) we take the parameters as

$$\begin{array}{cc} & \eta =0.8,\;\;\tau =1.5,\;\;D_{1}=0.002,\;\;D_{2}=0.08,\\ & u^{*}=0.3,\;\;v^{*}=52.73333334,\;\;\epsilon =240,\;\;\gamma =0.005689001264. \end{array}$$ (55)

Fig 5(b) is spirals 2.

In Fig 5(c) we take the parameters as

$$\begin{array}{cc} & \eta =0.85,\;\;\tau =1.5,\;\;D_{1}=0.01,\;\;D_{2}=0.38,\\ & u^{*}=0.3,\;\;v^{*}=19.13333333,\;\;\epsilon =80,\;\;\gamma =0.01567944251. \end{array}$$ (56)

Fig 5(c) is spirals 3.

## Discussion

In this paper, a spatial Lesile type predator-prey system with Holling type III functional response and time delay has been investigated. To well understand the impact of delay and diffusion on the instability, we have made theoretical analysis and numerical simulations. Since the equilibrium cannot be expressed in a useful closed form, we cannot discuss its qualitative properties in normal routine. Firstly, we discuss the number and qualitative properties of positive equilibrium via the original parameters. Secondly, we obtain conditions of two types of instability: diffusion induced instability and delay induced instability. Finally, numerical simulations are performed to illustrate the theoretical findings. Both the theoretical and numerical results reveal that the interaction between time delay and diffusion can give rise to stationary patterns.

However, it should be noted that the method in this paper is only suitable for short time delay *τ*. When the delay is large, one should use other methods to find the condition for Turing instability. Moreover, we may investigate travelling wave of model (5) in the future study. It should be also worth pointing that other types of instability may be found in system (5). For example, we can use normal formal theory and the center manifold theorem of partial functional differential equations to analyze the Hopf bifurcation of system (5) [3].

## Supporting Information

S1 File Qualitative properties and stability of the interior equilibria of system (3). (PDF)

## References

[1] Wollkind DJ, Collings JB, Logan JA. Metastability in a temperature-dependent model system for predator-prey mite outbreak interactions on fruit trees. Bull. Math. Biol. 1988;50(4):379–409.

[2] Hsu SB, Huang TW. Global Stability For A Class Of Predator-Prey Systems. Siam J. Appl. Math. 1995;55(3):763–783. 10.1137/S0036139993253201

[3] Sun GQ, Wang SL, Ren Q, Jin Z, Wu YP. Effects of time delay and space on herbivore dynamics: linking inducible defenses of plants to herbivore outbreak. Scientific Reports 2015;5:11246 10.1038/srep11246 26084812 PMC4471659

[4] Jones LE, Ellner SP. Evolutionary tradeoff and equilibrium in an aquatic predator-prey system. Bull. Math. Biol. 2004;66(6):1547–1573. 10.1016/j.bulm.2004.02.006 15522345

[5] Guan XN, Wang WM, Cai YL. Spatiotemporal dynamics of a Leslie-Gower predator-prey model incorporating a prey refuge. Nonlinear Analysis Real World Applications 2011;12(4):2385–2395. 10.1016/j.nonrwa.2011.02.011

[6] Sun GQ, Wu ZY, Wang Z, Jin Z. Influence of isolation degree of spatial patterns on persistence of populations. Nonlinear Dyanm. 2016;83:811–819. 10.1007/s11071-015-2369-6

[7] Jost C. Comparing predator-prey models qualitatively and quanti tatively with ecological time-series data. Ph.D. thesis. Paris Grignon: Institute National Agrronomique; 1998.

[8] Bazykin AD. Nonlinear Dynamics of Interacting Populations. Singapore: World Scientific Publishing, 1998.

[9] Leslie PH. Some further notes on the use of matrices in population mathematics. Biometrika 1948;35:213–245. 10.1093/biomet/35.3-4.213 21006835

[10] Leslie PH, Cower JC. The properties of a stochastic model for the predator-prey type of interaction between two species. Biometrika 1960;47:219–234. 10.1093/biomet/47.3-4.219

[11] Freedman HI, Mathsen RM. Persistence in predator-prey systems with ratio-dependent predator influence. Bull. Math. Biol. 1993;55:817–827. 10.1007/BF02460674

[12] Hsu SB, Huang TW. Global stability for a class of predator-prey system. SIAMJ. Appl. Math. 1995;55:763–783. 10.1137/S0036139993253201

[13] Segel L and Jackson J. Dissipative structure: an explanation and an ecological example. J. Theor. Biol. 1972;37:545–559. 10.1016/0022-5193(72)90090-2 4645361

[14] Sun GQ, Jin Z, Li L and Li BL. Self-organized wave pattern in a predator-prey model. Nonlinear Dynam. 2010;60:265–275. 10.1007/s11071-009-9594-9

[15] Levin S, Segel L. Hypothesis for origin of planktonic patchiness. Nature 1976;259(5545):659–659. 10.1038/259659a0814470

[16] Sun GQ, Jin Z, Liu QX, Li L. Dynamical complexity of a spatial predator-prey model with migration. Ecol. Model. 2008;219:248–255. 10.1016/j.ecolmodel.2008.08.009

[17] Sengupta A, Kruppa T, Lowen H. Chemotactic predator-prey dynamics. Phys. Rev. E 2011;83(3):1133–1144. 10.1103/PhysRevE.83.031914 21517532

[18] Sun GQ, Zhang J, Song LP, Jin Z, Li BL. Pattern formation of a spatial predator-prey system. Appl. Math. Comput. 2012;218:11151–11162. 10.1016/j.amc.2012.04.071

[19] Cushing JM. Integrodifferential equations and delay models in population dynamics. Heidelberg: Springer-Verlag, 1997.

[20] Kuang Y. Delay differential equations with application in population dynamics. New York: Academic Press, 1993.

[21] Nindjin AF, Aziz-Alaoui MA, Cadivel M. Analysis of a prdator-prey model with modified Leslie-Gower and Holling-type II schemes with time delay. Nonlinear Anal. Real World Appl. 2006;7:1104–1108. 10.1016/j.nonrwa.2005.10.003

[22] Xu R and Chen LS. Persistence and stability for a two-species ratio-dependent predator-prey system with time delay in a two-patch environment. Comput. Math. Appl. 2000;40(0):577–588.

[23] Yafia R, Adnani F, Alaoui H. Limit cycle and numerical simulations for small and large delays in a preydator-prey model with modified Lesile-Gower and Holling-type II schemes. Nonlinear Anal.: Real World Appl. 2008;9(5):2055–206. 10.1016/j.nonrwa.2006.12.017

[24] Ruan S. On nonlinear dynamics of predator-prey models with discrete delay. Math. Modelling Nature Phenom. 2009;4(2):140–188. 10.1051/mmnp/20094207

[25] Faria T. Stability and bifurcation for a delayed predator-prey model and the effect of diffusion. Math. Anal. Appl. 2001;254:433–463. 10.1006/jmaa.2000.7182

[26] Hadeler KP, Ruan S. Interaction of diffusion and delay. Discrete Contin. Dyn. Syst. B 2007;8:95–105. 10.3934/dcdsb.2007.8.95

[27] Yang XP. Stability and Hopf bifurcation for a delayed prey-predator system with diffusion effects. Appl. Math. Comput. 2007;192:552–566. 10.1016/j.amc.2007.03.033

[28] Yan S, Lian XZ, Wang WM. Spatiotemporal dynamics in a delayed diffusive predator model. Appl. Math. Comput. 2013;224:524–534. 10.1016/j.amc.2013.08.045

[29] Sun GQ, Chakrabort A, Liu QX, Jin Z, Anderson KE, Li BL. Influence of time delay and nonlinear diffusion on herbivore Outbreak. Commun. Nonlinear Sci. Numer. Simulat. 2014;19:1507–1518. 10.1016/j.cnsns.2013.09.016

[30] Sen S, Ghosh P, Riaz S, Ray D. Time-delay-induced instabilities in reaction-diffusion systems. Phys. Rev. E 2009;80(4):2016–2023. 10.1103/PhysRevE.80.046212 19905420

[31] Ghosh P. Control of the Hopf-Turing transitiion by time-delayed global feedback in a reaction-diffusion system. Phys. Rev. E 2011; 84(1):1183–1206. 10.1103/PhysRevE.84.016222 21867288

[32] Hu HX, Li QS and Li. Traveling and standing patterns induced by delay feedback in uniform oscillatory reaction-diffusion system. Chem. Phys. Lett. 2007;447(4):364–367. 10.1016/j.cplett.2007.09.031

[33] Li QS and Hu HX. Pattern transitions induced by delay feedback. J. Chem. Phys 2007;127(15). 10.1063/1.2792877 17949176

[34] Tang YL, Huang DQ, Zhang WN. Direct parametric analysis of an enzyme-catalyzed reaction model. IMA Joural of Applied Mathematics 2011;76(6):876–898. 10.1093/imamat/hxr005

[35] Sun GQ, Jin Z, Liu QX, Li L. Pattern formation in a spatial S-I model with non-linear incidence rates. J. Stat. Mech. 2007;11:1101.

[36] Sun GQ, Jin Z, Liu QX, Li L. Chaos induced by breakup of waves in a spatial epidemic model with nonlinear incidence rate. J. Stat. Mech. 2008;8:08011 10.1088/1742-5468/2008/08/P08011

[37] Aragón JL, Torres M, Gil D, Barrio RA, Maini PK. Turing patterns with pentagonal symmetry. Phys. Rev. E 2002;65(10):051913. 10.1103/PhysRevE.65.051913 12059599

[38] Sun GQ, Jin Z, Li L, Haque M, Li BL. Spatial patterns of a predator-prey model with cross diffusion. Nonlinear Dyn. 2012;69:1631–1638.

[39] Murray DJ. Mathematical Biology II: Spatial Models and Biomedical Applications. Berlin: Springer; 2003.

