# Supplementary material for: Spatial Patterns of a Predator-Prey System of Leslie Type with Time Delay
Source: PLoS One. 2016 Mar 1;11(3):e0150503. doi: 10.1371/journal.pone.0150503 (PMC4773104; doi:10.1371/journal.pone.0150503)
Supplement: S1 File — (PDF) [file pone.0150503.s001.pdf]

Spatial patterns of a predator-prey system of Leslie type with time delay

Caiyun Wang[1], Lili Chang[2], Huifeng Liu[3]
1 Department of Mathematics, Xinzhou Teachers University, Xinzhou 034000, Shan'xi, China
2 Complex Systems Research Center, Shanxi University, Taiyuan, Shanxi 030006, PR China
3 College of Material Science and Engineering, Taiyuan University of Science and Technology, Taiyuan 030024, Shan'xi, China

S1 File. Qualitative properties and stability of the interior equilibria of system (3).

Theorem 1. (i) If condition (13) and (14) hold, system (3) has three equilibria, the middle of which is a saddle but each of the others is either a node or a focus or of center type.
(ii) If condition (15) and (16) hold, system (3) has two equilibria, one of which is either a node, a focus or of center type while the other is degenerate.
(iii) If and only if (18), or (13) and (17) hold, system (3) has a unique equilibrium, which is either degenerate if $P = Q = 0$ and $-\omega_2^2/3 \in I_0$ or a node, a focus or of center type otherwise.

Proof. Let $E^*(u^*, v^*)$ be any equilibrium of system (3), where $v^* = \frac{u^*}{\gamma}$. The Jacobian matrix of system (3) at $E^*(u^*, v^*)$ takes the following form

$$J = \begin{pmatrix} \frac{\partial f}{\partial u} & \frac{\partial f}{\partial v} \\ \frac{\partial g}{\partial u} & \frac{\partial g}{\partial v} \end{pmatrix}_{(u^*,v^*)} \triangleq \begin{pmatrix} f_u & f_v \\ g_u & g_v \end{pmatrix} = \begin{pmatrix} \frac{-2\epsilon(u^*)^3-1+\epsilon(u^*)^2}{\epsilon(u^*)^2+1} & \frac{-(u^*)^2}{\epsilon(u^*)^2+1} \\ \frac{\eta}{\gamma} & -\eta \end{pmatrix}. \quad (1)$$

and the trace and determinant of matrix J are given by

$$\mathrm{tr}(J) = \frac{-2\epsilon(u^*)^3 + \epsilon(u^*)^2 - 1}{\epsilon(u^*)^2 + 1} - \eta \triangleq f_u + g_v, \quad (2)$$

$$\det(J) = \frac{\eta u^*}{\epsilon(u^*)^2 + 1} F'(u^*) \triangleq f_u g_v - f_v g_u. \quad (3)$$

Obviously the sign of $\det(J)$ is the same as $F'(u^*)$, for $\frac{\eta u^*}{\epsilon(u^*)^2+1} > 0$. It implies that the qualitative properties of E^* are decided by $\mathrm{tr}(J)$ and $F'(u^*)$. Namely, when E^* is degenerate if and only if u^* is a multiple root of F. When E^* is non-degenerate, it is a stable node (or focus), an unstable node (or focus) and of center type if $\mathrm{tr}(J) < 0, = 0, > 0$, respectively. By Fig. 1, Fig. 2, and Fig. 3, when system (3) has three equilibria, the middle one is a saddle for $F'(u^*)$ is negative there, but both of the others are either node, focus or of center type; when system (3) has two equilibria, one of which is degenerate but the other one is either a node, a focus or of center type because $F'(u^*) \neq 0$ there; when system (3) has a unique equilibrium, it is either a degenerate one if $P = Q = 0$ and $\frac{\omega_2}{3} \in I_0$ or one of three states such as node, focus and center type because $F'(u^*) >$ there. $\square$
